# Can the origin of biosynthetic routes be explained by a Frankenstein’s monster-like spontaneous assembly of prebiotic reactants?

**DOI:** 10.1098/rstb.2024.0289

**Published:** 2025-10-02

**Authors:** Alicia Negrón-Mendoza, Ricardo Hernández-Morales, Antonio Lazcano

**Affiliations:** ^1^ Instituto de Ciencias Nucleares, Universidad Nacional Autónoma de México, Ciudad de México 04510, Mexico; ^2^ Facultad de Ciencias, Universidad Nacional Autónoma de México, Ciudad de México 04510, Mexico; ^3^ El Colegio Nacional, Ciudad de México 06020, Mexico

**Keywords:** origin of metabolism, prebiotic chemistry, biosynthetic intermediates, abiotic assembly of metabolic pathways

## Abstract

Since it is easy to envision that prebiotically synthesized molecules underwent reactions comparable or even identical to those that occur in some metabolic pathways, it has been argued that biosynthetic routes emerged from the direct assembly of these prebiotic reactants. However, although in prebiotic simulation experiments several products present in extant metabolic routes may be formed at the same time, in biosynthetic pathways, the same precursor undergoes a stepwise transformation into other compounds, allowing changes of energy to occur that are necessary for the energetic independence of cells. In other words, from thermodynamic and equilibrium perspectives, the availability of many or all of the chemical components of a metabolic route does not necessarily imply that they will spontaneously be linked in linear or cyclic pathways. Although a number of chemical changes that mimic or are identical to a number of metabolic reactions may have taken place in the primitive environment, in the absence of a genetic apparatus ensuring the stability and diversification of the catalysts and hypothetical pathways, it is difficult to picture the continuity and evolution of such chains of reactions.

This article is part of the theme issue ‘Origins of life: the possible and the actual’.

## 1. Introduction

How did metabolic routes originate? The striking resemblance between many of the chemical constituents of present-day life forms and those found in extraterrestrial materials or synthesized in laboratory models of the primordial environment appears to be profound, and has been interpreted as indicating a period of chemical evolution as a necessary prerequisite for the emergence of life. Led by the widespread biological distribution of fermentative metabolism and its enzymes in both anaerobic and aerobic organisms [1,2], Oparin concluded that all living beings descended from an anaerobic heterotrophic ancestor. According to his original formulation, the growth and reproduction of the first living entities depended on the uptake of organic molecules of biochemical significance and other raw material present in the primitive environment [3–6].

Many complex chemical changes must have occurred in the primitive Earth. Did those changes prefigure the emergence of metabolic pathways? Experimental results and theoretical modelling of these abiotic processes have led to the idea that such abiotic transformations represent the primordial setting of protometabolic routes, i.e. that biochemistry recapitulates prebiotic chemistry. This includes, for instance, the possibility of an abiotic tricarboxylic acid cycle assumed to have originated on the surface of pyrite and other iron sulfide minerals [7,8], as well the related hypothesis of a reverse citric acid cycle suggested by Morowitz *et al*. [9]; see also [10].

In its more extreme form, some of these proposals assume that metabolism preceded replication, a possibility that in some cases represents a healthy rejection of the reductionist version of some formulations of the RNA world. However, as of today direct empirical evidence in support of the possibility that life started in the absence of a genetic apparatus is lacking. As far as we know, there is nothing in contemporary biological systems that indicates otherwise. The purpose of this paper is to analyse critically the likelihood that the origin of metabolic pathways lies in the chemical changes of components of the so-called primitive soup. The proposal that the emergence of metabolism did not require catalysts encoded by a primitive genetic system is also addressed, together with some alternatives to this idea.

## 2. Does biochemistry recapitulate prebiotic chemistry?

It has sometimes been argued [11] that the proposal that metabolism preceded a genetic system can be traced directly to the pioneering hypothesis of a heterotrophic origin of life suggested by Oparin a century ago [1,2]. This is a misinterpretation. As noted elsewhere, like many of his contemporaries, Oparin did not conceptualize heredity in terms of genes, but was a direct follower of Haeckel’s idea that both inheritance and metabolism resided in the so-called bacterial protoplasm. Like many others, he remained convinced of the incompatibility between Mendelism and Darwinian evolution, and Oparin continued to follow the idea that biological inheritance could be explained in terms of the growth and division of the bacterial protoplasm [4,6].

**Figure 1.**
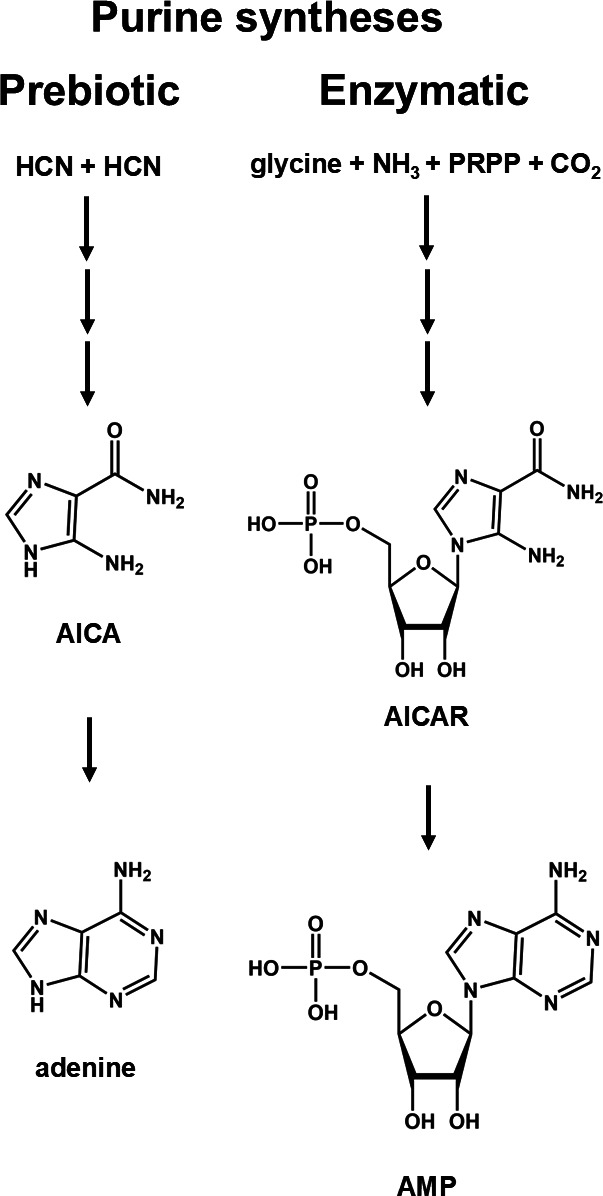
Schematic comparison of the abiotic (left side) and biological (right side) syntheses of purines. Both are multi-step processes that start with different precursors, but with the exception of the prebiotic intermediate 4-aminoimidazole-5-carbonitrile (AICA) and the biological ribotide equivalent (AICAR) they do not share any other similarity. PRPP, phosphoribosyl pyrophosphate.

The possibility that metabolic pathways resemble abiotic processes does have at least in part a long history. For instance, Oró [12] and Oró & Kimball [13] underlined that the abiotic synthesis of adenine from the pentamerization of HCN proceeds through the formation of 4-aminoimidazole-5-carbonitrile (AICA), which is of course similar to the aminoimidazole carboxamide ribotide derivative (AICAR), an intermediate in the biosynthesis of purines (figure 1). However, the similarity between the possible prebiotic synthesis of purines and their biosynthetic route is limited to this pair of compounds. This opens the possibility that the resemblance may be due solely to chemical determinism, and does not necessarily probe a relationship of descent between the two processes.

There are some additional examples that would seem to support the possibility that biochemistry recapitulates prebiotic chemistry, and differs solely by the intervention of enzymes which would have evolved afterwards. The list comprises some individual steps of metabolic pathways, including (i) the synthesis of pyrroles from δ-aminolaevulinic acid [14]; (ii) the alkaline degradation of glucose-6-phosphate [15]; (iii) the clay-mediated deamination of adenine [16]; (iv) the photochemical UV decarboxylation of orotic acid into uracil, which is analogous to the enzymatic decarboxylation of orotidylate [17]; and (v) the formation of orotic acid from photodehydrogenation of dihydroorate [18].

However, these examples do not necessarily qualify as evidence of protometabolic reactions. Some of these reactions were conducted under laboratory-controlled conditions that are not necessarily geologically plausible and, perhaps more importantly, may be explained by chemical determinism because owing to physicochemical restrictions they may be the only way in which a reaction, whether biological or abiotic, can take place. In fact, with few exceptions, there are major chemical differences between the extant biosynthetic pathways and abiotic processes. For instance, amino acids can be formed abiotically by the hydrolysis of HCN polymers or by the Strecker synthesis, but both processes are completely different from the biological enzyme-mediated transamination and the reverse carboxylic acid cycle (cf. [19]). These and many other examples suggest the absence of a simple continuity between the prebiotic and biosynthetic pathways.

The parallels between non-enzymatic reactions that are the same as in biosynthetic routes may be similar simply because they are mechanistically inevitable, and not because there is a relationship of descent between the two of them. Attempts to close the gap between top-down and bottom-up characterizations of the origin of metabolism have led to research proposals that depend on the combination of prebiotic systems or the laboratory development of novel ribozymes with highly conserved biological traits [20,21]. Such an approach may provide insights on the systems that could have facilitated the evolutionary emergence of elementary informational oligomers during primitive times, but it is difficult to picture their stability and diversification in the absence of a genetic apparatus.

## 3. Are metabolic pathways the outcome of self-assembly of prebiotic reactants?

It easy to accept that complex systems of purely physical and chemical nature must have played a role in the evolutionary transition between non-living and living systems. In spite of their intellectual fascination, non-equilibrium examples like the Belousov–Zhabotinsky reaction have little or no direct bearing on the origins of life. A notable example of a self-assembly phenomenon quite relevant to the emergence of living systems is the abiotic assembly of double lipid membrane systems, including vesicles, which do not require a system of genetic information transfer to form (cf. [22]). The list of self-assembly phenomena also includes: (i) self-assembly of nucleic acids (base-bearing polymers); (ii) formation of Fe–S catalytic clusters; (iii) the spontaneous formation of mineral and organic compound complexes, for instance clays and bases; and (iv) autocatalytic synthetic reactions, like the formose reaction [3,23–26].

Is it likely that in the absence of genetic systems a Frankenstein-like self-assembly of functional bits and pieces of prebiotic reactants led to the emergence of metabolism? Can equivalent phenomena be invoked to explain the emergence of biosynthetic pathways? In other words, can it be assumed that, in a manner somewhat equivalent to Dr Frankenstein’s revival of a functional human by putting together fragments, the precursors and intermediates of metabolic pathways available on the early Earth spontaneously assembled in pathways and continued to evolve, providing energy and carbon and nitrogen sources to early organisms?

We are firmly convinced that the answer to this question is negative. There are several examples of the simultaneous abiotic syntheses of several different metabolic intermediates that may be prebiotically relevant. These are the cases, for instance (i) of the production of several carboxylic acids that are intermediates of the carboxylic acid cycle from the gamma irradiation of aqueous solutions of acetic acid [27] and the irradiation of aqueous solutions of HCN and NH_4_CN [28,29], and (ii) of the abiotic glyoxylate reaction, which produces *α*-hydroxy acids and *α*-amino acids, as well as orotate [30]. For metabolic pathways to develop, however, what is important is not the mere synthesis and accumulation of organic compounds of biochemical significance, but the development of functional connections between the intermediates, followed by their persistence and evolution. Moreover, not all requisite components of the linear pathway or cycle are synthesized in the same experiment, for example the ketoacids implicated in the Krebs cycle. As argued here, it is somewhat unlikely that the functional chemical linkage spontaneously organized between the abiotically synthesized compounds could persist beyond the half-life of their components, and replicate and evolve in the absence of genetically coded catalysts, whether enzymes or ribozymes.

In other words, from thermodynamic and equilibrium perspectives, the mere availability of many or all of the chemical components of a metabolic route does not imply they are linked in metabolic networks. In biosynthetic pathways, the starter molecular precursor undergoes a stepwise transformation into other compounds, allowing changes of energy to occur, which are necessary for the energetic independence of cells. Of course, such vectorial processes will be limited if a number of side reactions take place [24]. These by-products may have included intermediates eventually linking the different modular subsystems that form core metabolism.

Bridging the gap separating the abiotic syntheses and accumulation of organic compounds and their changes and transformations from the oldest biochemical processes does not imply disregarding the limits and potential that physics and chemistry impose on organic molecules [3,31]. It is clear that the highly refined extant pathways are the historical outcome of the evolutionary amplification of the basic physico-chemical properties that their simpler predecessors possessed [5]. The same is true, of course, of sets of reactions involving prebiotically formed biochemical compounds. Prebiotic organic molecules quite likely underwent many complex transformations and may have spontaneously organized into not very many sequential steps. As of today, the two examples are the formose reaction, which is the only example of an autocatalytic cycle and, based on Eschenmoser proposed glyoxylose reaction, starting with glycolaldehyde from glyoxylate, followed by a sequence of repetitive aldol condensations with glyoxylate, carbonyl migrations and decarboxylations can lead to biologically relevant *α*-hydroxy acids, *α*-amino acids and orotate [30].

As reviewed by Peretó and Carbonell & Peretó [32,33], there are several examples of abiotic cycles that may be prebiotically significant. The list includes: (i) the suggestion of a simple cyclic synthesis of HCN tetramer from HCN in the presence of formaldehyde [34]; and (ii) the so-called ‘sugar model', based on the reaction between glyceraldehyde and NH_3_ in an aqueous solution. In this system, the production rate of pyruvaldehyde is enhanced when ammonia is subsequently introduced into a fresh glyceraldehyde solution [35]. However enticing, in the absence of genetically encoded catalysts, the persistence and evolution of these cycles would have been constrained by short timescales defined by the half-life of their components. In other words, complex, self-organized chemical systems of reactions are not adapted to ensure their own survival and reproduction. They just exist and, unless continuous sources of precursors and energy are provided, they will eventually degrade.

There is no indication that life could have evolved in the absence of a genetic replicating mechanism ensuring the stability and diversification of its basic components, including, of course, metabolic catalysts. The same is true if such hypothetical metabolic pathways depended on metals, clays or other catalysts available in the environment. This does not imply that the issue of the emergence of life can be reduced to the origin of genetic material, but a recognition that no sequence of chemical reactions, although efficient, will have by itself an adaptative value unless it is part of a more complex system that replicates with variation. In the absence of hereditary mechanisms, abiotic sequences of successive reactions would, at best, come and go without leaving any direct descendants able to resurrect the process. As underlined by Eschenmoser [36], metabolic cycles are not evolvable systems by themselves, but could have played a key role as contributors to the emergence of elementary informational oligomers.

## 4. The semi-enzymatic theory of the origin of metabolism: an update

Non-enzymatic reactions are uncommon in core and central metabolism [37]. Although the number of spontaneous reactions that are known to take place in extant biosynthesis pathways is quite small, their existence supports the possibility of semi-enzymatic metabolic pathways that may have existed during early stages of biological evolution once genetically encoded catalytic peptides or primitive proteins had evolved [19].

The inventory of spontaneous reactions that are part of metabolic pathways supports the possibility of a primordial enzyme-free set of primitive anabolic and catabolic pathways, and has led to laboratory models of non-enzymatic glycolysis and the pentose phosphate pathway [38,39] . An additional example is the reduction of the γ-carboxyl group that yields l-glutamate semialdehyde, which then undergoes a spontaneous cyclization reaction and produces Δ-proline-γ-semialdehyde, a five-membered ring compound that is then reduced and yields proline. Analysis of the inventory of metabolic non-enzymatic reactions shows that a number of cyclization reactions that take place via an Amadori rearrangement are conspicuous, as in the case of imidazole formation in the histidine route and in the autocatalytic biosynthesis of 4-methylideneimidazole-5-one (MIO), which takes place via the self-condensation of a simple Ala-Ser-Gly [40,41].

Not all pathways that include spontaneous reactions are equally ancient. This is the case for the biosynthesis of patellamide, a cyanobacterial peptide [42], and for betaxanthin formation, a plant pigment [43]. Several non-enzymatic and spontaneous reactions driven by UV light are listed in the KEGG database (https://www.genome.jp/kegg/), including the conversion of ergosterol into vitamin D2 and of 7-dehydrocholesterol into vitamin D3. Although steroids are a late evolutionary development, these reactions are proof-of-principle examples and support the likelihood of spontaneous reactions as part of older biochemical pathways that may have been more significant during the early stages of biological evolution.

In some cases, the corresponding chemical change can be achieved under enzyme-free laboratory conditions by altering the reaction conditions and reagents. For instance, the well documented non-enzymatic addition of nitrogen under high ammonia concentration to *N*^1^−58-phospho-ribulosylformimino-5-aminoimidazole-4-carboaxamide ribotide produces imidazole glycerol phosphate (IGP) (cf. [19]). Quite importantly, this can also occur in biological systems, as shown by the prototrophic growth of a *Klebsiella pneumoniae* strain with a mutated non-functional *hisH* gene under high ammonia concentrations [44].

The attempt to link enzyme-free spontaneous biochemical reactions with prebiotic syntheses led to the proposal of the semi-enzymatic theory of the origin of metabolism, which included the utilization of molecules leaking from preexisting pathways as well as prebiotic compounds present in the environment ([19]; see also [37]). The semi-enzymatic proposal is based in part on Horowitz's proposal of retrograde metabolic evolution [45,46], raising the possibility that in ancient times parts of the biosynthetic routes resulted from non-enzymatic reactions, followed by the evolutionary development of starter-type enzymes that were already genetically encoded. But what happened before the origin and early diversification of such enzyme starter types? Of course, one could assume the presence of prebiotically synthesized peptides or other chemical catalysts or cofactors, but even if we assume a highly efficient process accumulating these compounds, they would eventually be exhausted.

## 5. Ribozymes, modified ribonucleotides and early metabolism

As reviewed elsewhere [47], the catalytic properties of nucleotide coenzymes and the participation of RNA monomers in metabolic pathways led long ago to several independent proposals of protein-free primordial life forms in which ancestral metabolism was catalysed by ribonucleotidyl coenzymes [48–55].

Approximately 50% of metabolic enzymes require at least one coenzyme, many of which are nucleotides or ribonucleotidyl derivatives like thiamine pyrophosphate, tetrahydrofolate and pyridoxal phosphate, whose nucleobase moieties are derived from nucleotides [54–57]. However, participation of nucleic acid bases or nucleotide derivatives is not in itself a criterion that defines the antiquity of a coenzyme-mediated pathways. Uridine diphosphate glucose is a key intermediate in the biosynthesis of ascorbic acid, but the latter is clearly a later metabolic innovation, as shown by its key role in collagen biosynthesis, which could not develop prior to the availability of free oxygen (cf. [58]).

The evidence that coenzymes catalyse reactions similar to those in which they partake as enzyme cofactors includes, for instance the thiamine-mediated reactions of carbonyl compounds in high pH, protein-free systems [59] and the pyridoxal-mediated oxidative deamination of amino acids [60]. The list now includes enzyme-free transamination, nucleoside phosphorylation of nucleoside phosphates, and reduction of keto acids by pyridoxal 5′-phosphate, adenosine diphosphate and nicotinamide adenine dinucleotide hydride, respectively, all of which strongly support the likelihood of ancestral metabolic pathways mediated at least in part by ribozymes and ribonucleotide derivatives [61].

In its original formulation, the semi-enzymatic theory of metabolism assumed implicitly the previous development of genetically encoded starter types of enzymes, i.e. the existence of an older period of biological evolution separating genotype from phenotype [19]. It is possible to update the theory and argue, for instance, for older stages in which catalytic RNA molecules or chemically active ribonucleotide derivatives played a key role. In principle, this could have occurred in a hypothetical pre-DNA world in which RNA was the genetic polymer. The possible existence of metabolic reactions during an early epoch in which RNA and ribonucleotide derivatives where catalysts is supported by the extraordinary experimental expansion of the catalytic repertoire of *in vitro* selection of synthetic ribozymes [62] that catalyse the same classes of chemical reactions as enzymes (cf. [63]).

Although we tend to think of the RNA world as a system defined solely by catalytic and replicative RNAs, a more comprehensive view should include a wide range of molecules, including ribonucleotides and their derivatives, which play directly or indirectly manifold roles in different biological processes. These molecules are energy-carrying compounds, coenzymes and precursors of coenzymes, precursors of histidine, alarmones or precursors of alarmones and precursors of deoxyribonucleotides. In fact, the widespread presence of nucleotide coenzymes and modified ribonucleotides in metabolic pathways [52,53] can be seen as an indication of an early intertwining of genetic material and biochemical catalysis.

The involvement of RNA molecules and modified ribonucleotides in metabolism may go beyond the ribonucleotidyl coenzyme dependence of many metabolic enzymes. Together with cysteine present in Fe–S catalytic clusters, histidine is the most abundant catalytic amino acid residue in six of the functional classifications of the EC1 and EC2 classes of enzymes [64]. It has been suggested that histidine, which is the only amino acid whose biosynthesis starts with a ribonucleotide, may be a molecular vestige of a catalytic ribonucleotide dating from an earlier biochemical stage in which RNA played a central role in biological catalysis [54,55].

Well studied examples of interactions between different ribozymes, modified nucleotides and metabolites include the glmS riboswitch–ribozyme system, which self-cleaves when bound to glucosamine-6-phosphate (GlcN6P). Can the regulatory role of riboswitches and the interaction of ribozymes with metabolites [65] be seen as indicative of an earlier semi-ribozymic biochemical stage in which RNA and ribonucleotide derivatives, including complexes of catalytic RNA with metal ions or peptides [66], played a key role in metabolism?

Of course, the scarcity of evidence gives considerable freedom to speculations on the origin of life, and the existence of a primordial RNA world is a fiercely contested topic. It is true that the genetic, structural and catalytic properties of RNA, together with the wide distribution of modified ribonucleotides in basic cellular processes, are certainly consistent with the possibility of an RNA/protein world [63]. However, RNA molecules and their components are highly optimized molecules that may have been preceded by simpler pre-RNA genetic polymers or may have been derived, together with DNA, from a mixture of chimeric genetic polymers, including threose nucleic acid/RNA and RNA/DNA oligonucleotides [67,68].

**Figure 2.**
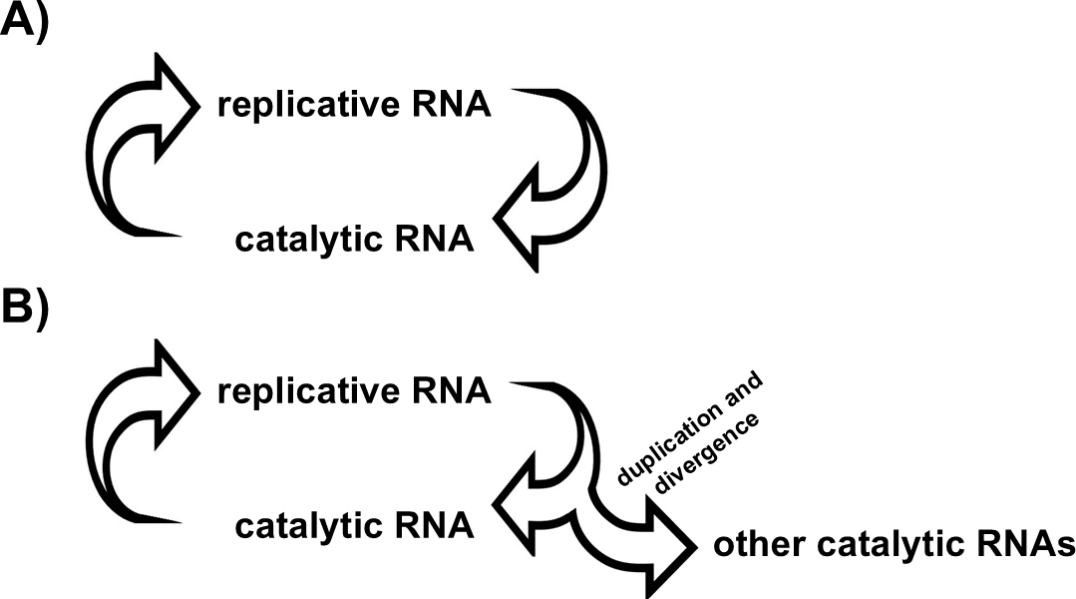
(A) A highly simplified scheme based on a reductionist model of the RNA world hypothesis formed by replicative and catalytic RNA molecules; (B) duplication and divergence of a sequence encoding a ribozyme could lead, in principle, to the recognition and modification of other substrates involved in primitive metabolic pathways.

As shown in figure 2, very simplistic schemes amenable to experimental analysis can be pictured in which an evolving genetic system encoding its own ribozymic polymerase undergoes a duplication event that diverges and evolves catalytic activities other than polymerization. Directed-evolution experiments have shown that adaptation to novel substrates typically involves deregulation and amplification of genes encoding enzymes lacking absolute substrate specificity [69]. These observations are consistent with the assumed properties of unregulated, primitive catalytic proteins with broad substrate specificity that may have existed during early stages of evolution [70]. The same may be true of ribozymes, which like enzymes exhibit a certain level of substrate and template promiscuity. Some of these additional reactions may have led to a diversification of RNA-mediated catalysis, including ancestral cofactors and simple metabolic-like reactions. However, a detailed account of the transition from a hypothetical primordial RNA world, in which RNA played a key role in primordial metabolism, to the semi-enzymatic metabolic state proposed by Lazcano & Miller [19] which may have inherited its ribonucleotidyl-cofactor dependence, will be incomplete until a solution to the problem of the origin of genetically encoded RNAs and peptides is found.

## 6. Conclusions

As argued here, it is likely that prebiotic organic compounds underwent many transformations, but given the differences from successive chemical changes that occur in metabolic pathways, perhaps we should refrain from thinking of them as ancestral to metabolism. How the latter emerged does not depend solely on the prebiotic availability of precursors and intermediates. The key difference from actual metabolism lies not in the chemical transformations themselves, but in the absence of a functional linkage between precursors, intermediates and end-products and, most importantly, in the absence of genetically encoded catalysts.

Even if all or many of the components and intermediates of metabolic cycles were available simultaneously and linked together by a succession of chemical transformations comparable to biosynthetic routes, there is no evidence that primitive metabolic cycles could spontaneously self-organize, persist and evolve in the absence of a genetic apparatus. Metabolic routes do not assemble themselves like the different functional bits and pieces of Dr Frankenstein's monster coming together. It is quite likely that the simultaneous abiotic syntheses of many of their individual components took place, but the pathways themselves are the historical outcome of evolutionary processes that must have required replicative systems capable of generating adaptative complexity by Darwinian processes in which selection operated not only over the individual genes but also over the sets of genetically encoded catalysts linked into functional units.

It is possible that the frequently invoked opposition between the origin of metabolism and the emergence of replication may be, in fact, a false dilemma. As noted above, the strict requirement of ribonucleotidyl coenzymes in metabolic pathways could be understood as an indication of an early intertwining of genetic material and biochemical catalysis [53]. As suggested by Eschenmoser [36], metabolic cycles are not evolvable systems by themselves, but could have played a key role as contributors to the emergence of elementary informational oligomers. The same is probably true of clay- or other mineral-based systems. How a functional relationship developed between metabolic cycles and replicative systems developed remains an open question, but as shown in figure 2, a partial answer based on a simplistic reductionist version of the RNA World lies in the duplication and diversification of catalytic polynucleotides. Of course, once encoded peptides emerged, the situation changed radically.

The original proposal of the heterotrophic origin of life assumed that the first organisms had formed from the prebiotically available organic molecules, that also became a source of carbon and energy. Nowadays perhaps a reformulation of the original theory would simply imply that primordial heterotrophy means direct uptake of organic compounds from the prebiotic environment, harnessing the intrinsic high-energy levels of compounds such as cyanamide, thioesters, glycine nitrile and other molecules (cf. [19]).

With the exception of oxygen-dependent metabolisms, it is not possible to assign a precise chronology to the development of biochemical pathways. However, the possibility of their rapid appearance is consistent with our current understanding of microbial population genetics, which shows that the acquisition of new metabolic traits does not necessarily follows a slow evolutionary pace. Such mode of evolution is consistent with the well-documented experimental selection of variants that develop in few days or weeks, the ability to use novel sources of carbon and nitrogen [69,71–75]. Such directed-evolution experiments, which may also provide insights on the development of anabolic pathways, have shown that adaptation to novel substrates typically involves deregulation and amplification of genes encoding enzymes lacking absolute substrate specificity [69]. These observations are consistent with the assumed properties of unregulated, primitive catalytic proteins with broad substrate specificity that may have existed during early stages of evolution.

Metabolism consists of individual components and systems of interacting molecules. Many of these subcellular units form modular structures that are functionally linked together. As time went by, their components and their interactions became increasingly more complex and refined. Components co-evolved because they were linked and encoded, i.e. did not change in isolation. In other words, the correlation between processes and components is the outcome of a historical process that, as suggested by the explosive metabolic evolution that apparently took place following the origins of life [76,77], took place very rapidly in evolutionary time.

Although there is a significant number of variations in metabolic pathways, the core of the basic genetic and molecular traits has persisted unchanged for over 3.5 Gyr. As underlined by Lazcano & Miller [78], this may be due to the linkage of the genes involved and the complex interactions between the different biosynthetic modules in which different routes are organized. This represents, in itself, a major case of biological conservatism that has been largely ignored.

## Data Availability

This article has no additional data.
